# CHRONIC CONDITIONS AND MORTALITY: MODERATION BY SELF-RATED HEALTH

**DOI:** 10.1093/geroni/igac059.1777

**Published:** 2022-12-20

**Authors:** Lauren Voss, Elizabeth Teas, Elliot Friedman

**Affiliations:** Purdue University, West Lafayette, Indiana, United States; Purdue University, Lafayette, Indiana, United States; Purdue University, West Lafayette, Indiana, United States

## Abstract

Chronic conditions become more common with age and greater numbers and severity of chronic conditions, in turn, increase the risk of mortality. However, individuals with similar disease burden often have different mortality rates. The purpose of the present study is to examine potential explanations for divergent mortality outcomes. Self-rated health (SRH), or perceptions of one’s own health, consistently predicts mortality. Thus, we hypothesized that participants’ SRH would modify the association between chronic conditions and mortality. Data were from the second wave of the Midlife in the US (MIDUS study, N=5,524). Mortality data were collected through 2018. Chronic conditions were measured in two different ways. To assess disease severity, each chronic condition was weighted by its propensity to cause disability; these were then summed. The second was a count of chronic conditions, a common measure in many studies. SRH was measured on a scale of 1-5 (1 = poor, 5 = excellent). Results from logistic regression models showed probability of mortality increased significantly with greater disease burden (measured both as counts and severity of conditions) and decreased with higher ratings of SRH. Importantly, compared to lower ratings, higher ratings on SRH were associated with lower probability of mortality at the same levels of disease severity. In fact, participants who rated their health as excellent showed no increase in probability of mortality with increasing number or severity of conditions. Overall, this study suggests that even in the context of chronic diseases, positive perceptions of health predict greater longevity.

